# Multidimensional and Physical Frailty in Elderly People: Participation in Senior Organizations Does Not Prevent Social Frailty and Most Prevalent Psychological Deficits

**DOI:** 10.3389/fpubh.2020.00276

**Published:** 2020-07-21

**Authors:** Magdalena Sacha, Jerzy Sacha, Katarzyna Wieczorowska-Tobis

**Affiliations:** ^1^Department of Palliative Medicine, Poznan University of Medical Sciences, Poznaǹ, Poland; ^2^Faculty of Physical Education and Physiotherapy, Opole University of Technology, Opole, Poland; ^3^Department of Cardiology, University Hospital, University of Opole, Opole, Poland

**Keywords:** frailty, multidimensional frailty, non-robust, frail scale, Tilburg Frailty Indicator

## Abstract

**Purpose:** The study explores how the involvement in community-based senior organizations affects the prevalence of multidimensional and physical frailty among community dwelling elderly people.

**Materials and Methods:** The group of 1,024 elderly people (270 males) over the age of 65 years (mean age 72.6 ± 6.3 years; range 65–93 years) took part in this study. The subjects completed a questionnaire regarding multidimensional (i.e., the Tilburg Frailty Indicator, TFI) and physical frailty (i.e., the FRAIL scale), as well as factors associated with frailty and participation in senior organizations.

**Results:** The prevalence of multidimensional frailty (if at least 5 points in the TFI) was 54.6%, and the prevalence of physical frailty (if at least 3 points in the FRAIL scale) and a non-robust status (if any point in the FRAIL scale was positive) was 6.3 and 52.9%, respectively. The most prevalent frailty deficits were missing other people (66.6%), feeling nervous or anxious (65.9%), and feeling down (65.5%). Members of senior organizations presented a lower prevalence of multidimensional and physical frailty comparing with non-members. This was mainly caused by a lower prevalence of physical deficits and problems with memory; however, the prevalence of social deficits was similar in both groups. Senior organizations had no influence on the most widespread frailty deficits, i.e., missing other people, feeling nervous or anxious, and feeling down.

**Conclusions:** Multidimensional frailty and physical non-robust status are common among people over the age of 65 years. Participation in senior organizations is associated with lower risk of physical frailty; however, it has no effect on social frailty and the most prevalent psychological deficits. This information has important implications for practical management with senior problems and may influence community strategies concerning elderly people.

## Introduction

Since the human society is aging, age-related problems are becoming more and more prevalent in the general population (1). Frailty is one such problem associated with the ongoing demographic changes, and it constitutes a significant burden for both health and social systems as well as national economies (2, 3). Frailty is usually considered as a set of physical deficits causing a decrease in overall reserve capacity; however, it can also concern psychological and social domains of human functioning (4–7). Deficits in any of these domains may result in deficits in another, and their interactions accelerate functional decline of the elderly people. Therefore, an approach to frailty should be multidimensional (i.e., physical, psychological, and social) because such a concept more adequately reflects a complexity of the decrease in physiological reserves associated with aging (6, 8, 9). Moreover, the elderly people may present different types of functional deterioration and hence require an individualized approach to ensure their independence and good functioning (10).

Community-based senior organizations are considered to be a platform which may increase physical activities among elderly people, their social connections, and their mental functions. Such organizations gather people in a social atmosphere in their community-based meeting points and may help in promotion and realization of various healthy projects (11). However, despite all these potentials, little is known about how the participation in these organizations influences the prevalence of frailty and, particularly, its impact on different frailty dimensions.

This study explores how the involvement in community-based senior organizations affects the frailty prevalence as well as physical, psychological, and social domains of human functioning among community dwelling elderly people.

## Materials and Methods

### Participants

People at the age over 65 years living in a community in Opole District (southwest Poland) were considered for this cross-sectional study. The subjects completed a questionnaire regarding multidimensional and physical frailty, as well as factors associated with frailty and participation in senior organizations. The questionnaire was anonymous and contained short information on the study purpose as well as rationale; the study protocol was approved by the Ethics Committee at the Poznan University of Medical Sciences. Questionnaires were distributed during healthy lifestyle promoting meetings organized by local community-based senior organizations in the period December 2017 and December 2018. These open-access meetings were dedicated to all elderly people living in a region (not only to the organizations members) and they were advertised by appropriate posters. Details on the data collection have been described elsewhere (10).

Multidimensional frailty has been evaluated using part B of the Tilburg Frailty Indicator (TFI) which contains 15 frailty components arranged according to three different domains. The physical domain (0–8 points) consists of eight items related to poor physical health, unintentional weight loss, difficulty in walking, difficulty in maintaining balance, poor hearing, poor vision, lack of strength in hands, and physical tiredness. The psychological domain (0–4 points) comprises four components related to problems with memory, feeling down, feeling nervous or anxious, and inability to cope with problems. The social domain (0–3 points) consists of three elements associated with living alone, missing other people, and lack of support from other people. The TFI total score may rank from 0 to 15; by definition, frailty is established if the TFI score is at least 5 (9). Part A of TFI is the basis for the assessment of risk factors predisposing to frailty which includes age, gender, education level, economic status, lifestyle, marital status, experiences with different unfavorable events in recent time, and satisfaction with living conditions (9). The TFI has been adapted and validated for Polish population by Uchmanowicz et al. (12, 13).

Physical frailty has been assessed with the help of the FRAIL scale which includes five components related to physical tiredness/fatigue, inability to walk up one flight of stairs, inability to walk 200 m, unexplained body mass loss, and a number of chronic diseases (14, 15). Unexplained body mass loss is scored 1 if respondents report their weight decline of 6 kg or more during the last 6 months, or 3 kg or more during the last month. The presence of 5 or more chronic diseases yields score 1; otherwise, it is scored 0. FRAIL scale scores range from 0–5 and may represent frail (3-5 points), pre-frail (1-2 points), and robust (0 points) status (14, 15).

The subjects were also asked about a place of living (city or village), former occupation (intellectual or physical one), and whether they participated in community-based senior organizations.

### Community-Based Senior Organizations

In Poland, senior organizations are financed either by local government or by non-profit organizations, and they are run by seniors themselves; in addition, they may be supported by workers of local social centers. These organizations integrate elderly people from different societies and generations, and their goal is to motivate seniors to take active part in the local social life, establish new connections, and spend time together. Such organizations promote healthy lifestyle, particularly encourage seniors to increase their physical and mental activities, propagate culture and art, and provide courses concerning computer technologies and diverse domains of knowledge as well as language courses. They organize trips, social meetings, theater and movie shows, as well as meetings with various experts and personalities. The organizations cooperate with each other and they also establish international cooperation. Activities are scheduled monthly and they are announced on websites and local meeting points. Memberships in these organizations is voluntary and usually free of charge.

### Statistical Analysis

Descriptive data are presented as mean ± standard deviation (SD) or frequency and percentage as appropriate. The normality of the variables was tested using the Kolmogorov–Smirnov test and visual inspection of histograms. Although most variables did not reveal a normal distribution, they were presented as mean ± SD to enable numerical comparisons. Differences between variables were compared using the Mann-Whitney-*U*-test or the Fisher's exact test. Independent determinants of participation in community-based senior organizations were identified with logistic regression through multiple testing. Variables with *p* > 0.1 in adjusted analyses were not retained in the final model. Determination coefficient (*R*^2^) and area under curve (AUC) were calculated for the regression model. The threshold probability of *p* < 0.05 was taken as the level of statistical significance. All analyses were performed using NCSS 12 Statistical Software (2018), NCSS, LLC, Kaysville, Utah, USA, ncss.com/software/ncss.

## Results

### Participants Characteristics

The study group comprised 1,024 participants over the age of 65 years (mean age 72.6 ± 6.3 years; range 65–93 years) and 270 were males. Most of the subjects presented a high school education level and a moderate economic status, they usually followed a partially healthy or healthy lifestyle, and 44.9% of them participated in senior organizations. A majority of them lived in a city and previously had intellectual occupations, half of them lived in relationships, some of them experienced various events and diseases; however, 88.2% declared satisfaction with living conditions. The detailed characteristics including frailty components are given in Table 1. The prevalence of multidimensional frailty (i.e., if at least 5 points in the TFI) was 54.6%, the prevalence of physical frailty (i.e., if at least 3 points in the FRAIL scale) was 6.3%, and a non-robust status (i.e., if any point in the FRAIL scale was positive) was diagnosed in 52.9%. Among all frailty components, those with the highest prevalence were missing other people (66.6%), feeling nervous or anxious (65.9%), and feeling down (65.5%).

**Table 1:** Study group characteristics.

**Characteristic**	**Overall group (*N* = 1,024)**
Age	72.6 ± 6.3
Male sex	270 (26.4)
Primary school education level	258 (25.2)
High school education level	464 (45.3)
University education level	302 (29.5)
Low economic status	152 (14.8)
Moderate economic status	835 (81.5)
High economic status	37 (3.6)
Unhealthy lifestyle	54 (5.3)
Partially healthy lifestyle	532 (52.0)
Healthy lifestyle	438 (42.8)
Participation in a senior organization	460 (44.9)
Living in a city	746 (72.9)
Living in a relationship	529 (51.7)
Former intellectual occupation	646 (63.1)
Death of a loved person in the recent time	389 (38.0)
Serious illness in the recent time	229 (22.4)
Serious illness of a loved person in the recent time	245 (23.9)
End of an important relationship in the recent time	70 (6.8)
Traffic accident in the recent time	59 (5.8)
Criminal event in the recent time	23 (2.2)
Satisfaction with living conditions	903 (88.2)
*The Tilburg Frailty Indicator (TFI)*	
1. Poor physical health	331 (32.3)
2. Unexplained body mass loss	133 (13.0)
3. Difficulty in walking	371 (36.2)
4. Difficulty in maintaining balance	261 (25.5)
5. Poor hearing	358 (35.0)
6. Poor vision	414 (40.4)
7. Lack of strength in hands	283 (27.6)
8. Physical tiredness/fatigue	465 (45.4)
9. Problems with memory	138 (13.5)
10. Feeling down	671 (65.5)
11. Feeling nervous or anxious	675 (65.9)
12. Inability to cope with problems	188 (18.4)
13. Living alone	384 (37.5)
14. Missing other people	682 (66.6)
15. Lack of support from other people	185 (18.1)
Sum of physical deficits (components: 1–8)	2.6 ± 2.1
Sum of psychological deficits (components: 9–12)	1.6 ± 1.1
Sum of social deficits (components: 13–15)	1.2 ± 0.9
Total score of TFI (all components)	5.4 ± 3.1
Multidimensional frailty according to TFI	559 (54.6)
*The FRAIL scale*	
1. Physical tiredness/fatigue	465 (45.4)
2. Inability to walk up one flight of stairs	87 (8.5)
3. Inability to walk 200 m	101 (9.9)
4. Unexplained body mass loss	133 (13.0)
5. Number of chronic diseases	1.9 ± 1.6
Total score for physical frailty according to the FRAIL scale	0.8 ± 0.9
Physical frailty according to the FRAIL scale	64 (6.3)
Non-robust status according to the FRAIL scale	542 (52.9)

*Values are n (%) or mean ± SD*.

### Participation in Community-Based Senior Organizations

Table 2 presents differences between members and non-members of senior organizations. Subjects participating in senior groups were slightly younger and presented a higher education level and economic status, a majority of them lived in a city and had a former intellectual occupation; they less commonly experienced the death of a loved person, serious illness, or a traffic accident, and they also declared higher satisfaction with living conditions. In terms of TFI, the individuals from senior groups revealed a lower prevalence of all physical components (items 1–8) and problems with memory (item 9); however, they more often lived alone (item 13). Consequently, they presented a lower rate of multidimensional frailty comparing with non-members of senior groups, i.e., 44.1 vs. 63.1%, *p* < 0.0001, respectively. This was mainly caused by a lower number of physical deficits and memory problems. However, the sum of social deficits and the prevalence of the most common frailty components (i.e., missing other people, feeling nervous or anxious, and feeling down) were not different in both groups (Table 2). According to the FRAIL scale, members of senior groups presented lower levels of physical tiredness and a smaller number of chronic diseases; in addition, they less frequently reported an inability to walk up one flight of stairs or 200 m, and an unexplained body mass loss. Consequently, physical frailty was significantly less frequent in members than in non-members of senior organizations (3.0 vs. 8.9%, p < 0.001), while a non-robust status accounted for 39.3 vs. 64%, *p* < 0.0001, respectively.

**Table 2:** Comparison of members and non-members of senior organizations.

**Characteristic**	**Members of senior organizations (*N* = 460)**	**Non–members of senior organizations (*N* = 564)**	***P*–value**
Age	**71.6** **±** **5.8**	**73.5** **±** **6.6**	**<0.0001**
Male sex	108 (23.5)	162 (28.7)	0.06
Primary school education level	**61 (13.3)**	**197 (34.9)**	**<0.0001**
High school education level	**241 (52.4)**	**223 (39.5)**	**<0.0001**
University education level	**158 (34.3)**	**144 (25.5)**	**<0.01**
Low economic status	**45 (9.8)**	**107 (19.0)**	**<0.0001**
Moderate economic status	**399 (86.7)**	**436 (77.3)**	**<0.001**
High economic status	16 (3.5)	21 (3.7)	0.86
Unhealthy lifestyle	24 (5.2)	30 (5.3)	0.94
Partially healthy lifestyle	250 (54.3)	282 (50.0)	0.17
Healthy lifestyle	186 (40.4)	252 (44.7)	0.17
Participation in a senior organization	–	–	–
Living in a city	**370 (80.4)**	**376 (66.7)**	**<0.0001**
Living in a relationship	231 (50.2)	298 (52.8)	0.41
Former intellectual occupation	**343 (74.6)**	**303 (53.7)**	**<0.0001**
Death of a loved person in the recent time	**144 (31.3)**	**245 (43.4)**	**<0.001**
Serious illness in the recent time	**76 (16.5)**	**153 (27.1)**	**<0.001**
Serious illness of a loved person in the recent time	100 (21.7)	145 (25.7)	0.14
End of an important relationship in the recent time	26 (5.7)	44 (7.8)	0.19
Traffic accident in the recent time	**19 (4.1)**	**40 (7.1)**	**<0.05**
Criminal event in the recent time	14 (3.0)	9 (1.6)	0.13
Satisfaction with living conditions	**423 (92.0)**	**480 (85.1)**	**<0.001**
*The Tilburg Frailty Indicator (TFI)*			
1. Poor physical health	**106 (23.0)**	**225 (39.9)**	**<0.0001**
2. Unexplained body mass loss	**45 (9.8)**	**88 (15.6)**	**<0.01**
3. Difficulty in walking	**116 (25.2)**	**255 (45.2)**	**<0.0001**
4. Difficulty in maintaining balance	**74 (16.1)**	**187 (33.2)**	**<0.0001**
5. Poor hearing	**115 (25.0)**	**243 (43.1)**	**<0.0001**
6. Poor vision	**147 (32.0)**	**267 (47.3)**	**<0.0001**
7. Lack of strength in hands	**84 (18.3)**	**199 (35.3)**	**<0.0001**
8. Physical tiredness/fatigue	**154 (33.5)**	**311 (55.1)**	**<0.0001**
9. Problems with memory	**36 (7.8)**	**102 (18.1)**	**<0.0001**
10. Feeling down	297 (64.6)	374 (66.3)	0.57
11. Feeling nervous or anxious	303 (65.9)	372 (66.0)	0.97
12. Inability to cope with problems	75 (16.3)	113 (20.0)	0.13
13. Living alone	**190 (41.3)**	**194 (34.4)**	**<0.05**
14. Missing other people	316 (68.7)	366 (64.9)	0.2
15. Lack of support from other people	73 (15.9)	112 (19.9)	0.1
Sum of physical deficits (components: 1–8)	**1.8** **±** **1.9**	**3.1** **±** **2.1**	**<0.0001**
Sum of psychological deficits (components: 9–12)	**1.5** **±** **1.0**	**1.7** **±** **1.1**	**<0.05**
Sum of social deficits (components: 13–15)	1.3 ± 0.9	1.2 ± 0.9	0.23
Total score of TFI (all components)	**4.6** **±** **2.8**	**6.0** **±** **3.2**	**<0.0001**
Multidimensional frailty according to TFI	**203 (44.1)**	**356 (63.1)**	**<0.0001**
*The FRAIL scale*			
1. Physical tiredness/fatigue	**154 (33.5)**	**311 (55.1)**	**<0.0001**
2. Inability to walk up one fight of stairs	**17 (3.7)**	**70 (12.4)**	**<0.0001**
3. Inability to walk 200 m	**16 (3.5)**	**85 (15.1)**	**<0.0001**
4. Unexplained body mass loss	**45 (9.8)**	**88 (15.6)**	**<0.01**
5. Number of chronic diseases	**1.7** **±** **1.5**	**2.0** **±** **1.6**	**<0.05**
Total score for physical frailty according to the FRAIL scale	**0.5** **±** **0.8**	**1.0** **±** **1.0**	**<0.0001**
Physical frailty according to the FRAIL scale	**14 (3.0)**	**50 (8.9)**	**<0.001**
Non-robust status according to the FRAIL scale	**181 (39.3)**	**361 (64.0)**	**<0.0001**

*Values are n (%) or mean ± SD. Statistically significant differences and p-values are marked in bold*.

Logistic regression analysis revealed several independent determinants for participation in the organized senior groups (Table 3). A high school and university education level as well as living in a city positively determined membership in the senior groups. Subjects were also more likely to belong to these groups if they experienced a criminal event in recent time or missed other people. On the other hand, a healthy lifestyle, living in a relationship, death of a loved person, serious illness, difficulty in maintaining balance, poor hearing, physical tiredness, and a lack of support from other people decreased the likelihood of being involved in senior organizations (Table 3).

**Table 3:** Determinants of participation in community-based senior organizations.

**Independent variables**	**Participation in senior organizations** ***R***^****2****^ **=** **0.22. AUC** **=** **0.75** ***p*** **<** **0.00001**	
	**B (SE)**	***p*–value**
Age		
Male sex		
High school education level	**1.03 (0.19)**	**<0.00001**
University education level	**0.88 (0.21)**	**<0.0001**
Moderate economic status		
High economic status		
Partially healthy lifestyle		
Healthy lifestyle	**−0.71 (0.33)**	**<0.05**
Participation in a senior organization	–	–
Living in a city	**0.59 (0.17)**	**<0.001**
Living in a relationship	**−0.52 (0.15)**	**<0.001**
Former intellectual occupation		
Death of a loved person in the recent time	**−0.74 (0.15)**	**0.00001**
Serious illness in the recent time	**−0.35 (0.18)**	**<0.05**
Serious illness of a loved person in the recent time		
End of an important relationship in the recent time		
Traffic accident in the recent time		
Criminal event in the recent time	**1.04 (0.48)**	**<0.05**
Satisfaction with living conditions		
*The Tilburg Frailty Indicator (TFI)*		
1. Poor physical health	331 (32.3)	
2. Unexplained body mass loss		
3. Difficulty in walking		
4. Difficulty in maintaining balance	**−0.47 (0.18)**	**<0.01**
5. Poor hearing	**−0.57 (0.15)**	**<0.001**
6. Poor vision		
7. Lack of strength in hands		
8. Physical tiredness/fatigue	**−0.42 (0.15)**	**<0.01**
9. Problems with memory		
10. Feeling down		
11. Feeling nervous or anxious		
12. Inability to cope with problems		
13. Living alone		
14. Missing other people	**0.41 (0.15)**	**<0.01**
15. Lack of support from other people	**−1.07 (0.31)**	**<0.001**
*The FRAIL scale*		
1. Physical tiredness/fatigue	**−0.42 (0.15)**	**<0.01**
2. Inability to walk up one flight of stairs		
3. Inability to walk 200 m		
4. Unexplained body mass loss		
5. Number of chronic diseases		

*Statistically significant coefficients and p-values are marked in bold*.

## Discussion

In this study, multidimensional frailty was present in 54.6% of 1,024 subjects over the age of 65 years and 6.3% revealed physical frailty, whereas 52.9% were non-robust (i.e., either physically frail or pre-frail). A concept which is intended to solve some of the deficits associated with aging, and consequently, decrease the prevalence of frailty, is a community-based senior organization (11). A wide range of initiatives of such organizations may potentially cover various types of seniors' needs; however, little is known about their impact on different frailty dimensions and which factors encourage people to participate in such initiatives. In this study, subjects who took part in senior organizations presented lower prevalence of all physical frailty deficits in both TFI and the FRAIL scale, as well as had fewer memory problems, yet, they more frequently lived alone. As a result, elderly people associated with senior organizations presented significantly lower prevalence of multidimensional frailty, physical frailty, and non-robust status. Several independent determinants for the participation in senior organizations have been identified, but it is not clear which of these variables are causes and which are effects of the involvement in senior groups. Indeed, numerous physical deficits may preclude elderly people from active participation in the organized senior life, e.g., poor hearing may prohibit them from establishing social connections. On the other hand, it is possible that the involvement in senior communities may motivate elderly people to a higher physical activity, and consequently, such a cohort presents lower physical frailty. However, despite these favorable effects in the physical domain, no such positive outcomes were observed in terms of social domain; in other words, social deficits were not less prevalent, and the sum of these deficits was not smaller in organized senior groups. Poor social relationships are some of the main reasons for low quality of life among elderly people (16–19). Moreover, for frail older individuals social contact is the most important factor for their life, while non-frail subjects consider health as the most critical one (17). It has been shown that loneliness is an independent determinant for functional decline and mortality in old age (20, 21). Thus, social factors are paramount in elderly populations; however, the present form of senior organizations seems to be ineffective in improving social life. In addition, senior organizations also had no influence on the most prevalent psychological deficits, i.e., feeling down and feeling nervous or anxious. In the report by Gobbens and van Assen (19), feeling down was the only frailty component that had an effect on all aspects of quality of life. Therefore, psychological problems seem to constitute the biggest challenge in dealing with the elderly people. Indeed, aging is associated with an unavoidable awareness of elapsing time, which reminds elderly people of the approaching end of life and significantly affects their psychological condition (7). Depression appears to be an ingredient of the aging process, and hence, it should be early recognized and appropriately treated, giving a chance to improve people's mood and motivation for their active life (22–24). Loneliness and a lack of occupation may lead to the loss of the life purpose which is a key element to activate elderly individuals. It has been shown that a stronger purpose in life among subjects over 50 years is associated with a decreased all-cause and some cause-specific mortality (25). Purposeful living may potentially improve both social and psychological domains of individual's functioning, and thus, this should be a task for community-based senior organizations. Moreover, a life purpose is a correctable risk factor and as such may be subjected to interventions. There are some data indicating that the prevention of physical frailty may defer psychological (cognitive) frailty, and this aspect should also be considered by senior organizations in planning an appropriate management (26, 27). Circumstances and external stimuluses may involve some activities in the elderly people provided that the intensity of these factors does not cross the limits of their capabilities. The necessity to deal with daily needs and various kinds of problems may extract some layers of energy in the elderly people, and it is connected to the aforementioned purposeful life and having a task in life. This is directly related to the idea of aging-in-place, the ideology promoted worldwide by the World Health Organization (28–32). Senior organizations may constitute a critical ingredient for this ideology; however, the present study shows that social and psychological aspects are not sufficiently covered by the current form of these organizations.

Since societies are aging, problems of social frailty along with psychological (or cognitive) frailty will be growing; hence, public awareness of these problems and an adequate adjustment of the activities of senior organizations are essential to abort or defer the functional elderly degradation. In fact, the approach to seniors should be individualized to their needs. Recently, it has been shown that simultaneous employment of TFI and the FRAIL scale may identify elderly people who require different managements, i.e., subgroups presenting predominantly social and psychological frailty and those with mainly physical deficits (10). These simple tools can be used by both seniors themselves or professionals in senior organizations to detect particular needs and plan an individualized management strategy. Moreover, these instruments may be utilized for monitoring the effects of senior organization activities. Indeed, such quality measures of social, psychological, and physical effects in subjects participating in the senior organizations may reflect their real value and help to improve their effectiveness.

## Study Limitations

The present study has, however, some limitations which should be acknowledged. First, we used self-reported questionnaires which were distributed among elderly people attending meetings dedicated for healthy lifestyle promotion, and therefore, some selection bias cannot be ruled out. Particularly, the participants were predominantly females, which is presumably due to the fact that the meetings probably attracted more females than males. Second, the cross-sectional nature of this study does not permit adequate cause-effect interpretations of the associations between various variables (i.e., the relationships between frailty deficits and the involvement in senior organizations). Third, although the FRAIL scale has been validated as a tool for quick diagnosis of physical frailty, whereas TFI for diagnosis of multidimensional frailty, they do not provide data coming from direct measurements of physical performance.

## Conclusions

In the community dwelling elderly people over the age of 65 years, more than one half present multidimensional frailty which corresponds to a similar percentage of non-robust subjects (i.e., physically frail or pre-frail). Participation in community-based senior organizations is associated with lower risk of physical frailty, yet, it has no effect on social frailty and the most common psychological problems. Social and psychological deficits are common among elderly people; however, since the present concept of senior organizations seems to be ineffective in solving these problems, some measures should be undertaken in order to adjust the activities of such organizations for the needs of elderly people. These observations have important implications for practical management with senior problems and may influence community strategies concerning elderly people.

## Data Availability Statement

The datasets generated for this study are available (for scientific cooperation) on request to the corresponding author.

## Ethics Statement

The studies involving human participants were reviewed and approved by Komisja Bioetyczna przy Uniwersytecie Medycznym w Poznaniu Ul. Bukowska 70, 60-812 Poznan. Written informed consent for participation was not required for this study in accordance with the national legislation and the institutional requirements.

## Author Contributions

MS, JS, and KW-T contributed to the study design, analysis and interpretation of data for the manuscript, critically revised the manuscript for important intellectual content, and approved the final version of the manuscript to be published. MS collected the data. MS and JS drafted the manuscript. All authors contributed to the article and approved the submitted version.

## Conflict of Interest

The authors declare that the research was conducted in the absence of any commercial or financial relationships that could be construed as a potential conflict of interest.
